# Upregulation of UBR1 m6A Methylation by METTL14 Inhibits Autophagy in Spinal Cord Injury

**DOI:** 10.1523/ENEURO.0338-22.2023

**Published:** 2023-06-02

**Authors:** Changsheng Wang, Xitian Zhu, Rongsheng Chen, Xiaobo Zhang, Nancheng Lian

**Affiliations:** Department of Spinal Surgery, First Affiliated Hospital of Fujian Medical University, Fujian, Fuzhou 350005, People’s Republic of China

**Keywords:** apoptosis, autophagy, m6A methylation, METTL14, spinal cord injury, UBR1

## Abstract

Gene Expression Omnibus database shows significantly downregulated expression of ubiquitin protein ligase E3 component N-recognin 1 (UBR1) in spinal cord injury (SCI). In this study, we investigated the mechanism of action of UBR1 in SCI. Following the establishment of SCI models in rats and PC12 cells, Basso–Beattie–Bresnahan (BBB) score and hematoxylin-eosin (H&E) and Nissl staining were used to evaluate SCI. The localization of NeuN/LC3 and the expression of LC3II/I, Beclin-1, and p62 were detected to assess autophagy. The expression of Bax, Bcl-2, and cleaved caspase-3 was detected and TdT-mediated dUTP-biotin nick end-labeling staining was employed to determine the changes in apoptosis. The N(6)-methyladenosine (m6A) modification level of UBR1 was analyzed by methylated RNA immunoprecipitation, and the binding of METTL14 and UBR1 mRNA was analyzed by photoactivatable ribonucleoside-enhanced crosslinking and immunoprecipitation. UBR1 was poorly expressed, and METTL14 was highly expressed in rat and cell models of SCI. UBR1 overexpression or METTL14 knock-down enhanced motor function in rats with SCI. Moreover, this modification increased Nissl bodies and autophagy and inhibited apoptosis in the spinal cord of SCI rats. METTL14 silencing reduced the m6A modification level of UBR1 and enhanced UBR1 expression. Importantly, UBR1 knock-down nullified METTL14 knock-down-induced autophagy promotion and apoptosis reduction. The METTL14-catalyzed m6A methylation of UBR1 promoted apoptosis and inhibited autophagy in SCI.

## Significance Statement

This study offers compelling evidence for METTL14-mediated m6A modification of UBR1 mRNA in modulating neuron autophagy and apoptosis in spinal cord injury (SCI), which may provide novel targets for modern SCI therapies.

## Introduction

Spinal cord injury (SCI) is a destructive neurologic insult which manifests as a series of pathologic events including ischemia, oxidative stress, inflammation, apoptosis, and locomotor dysfunction (Anjum et al., 2020). Primary injury is generated by an acute external physical impact and initiates a complex secondary injury that cyclically causes neuronal death and subsequent changes in the structural architecture of the spinal cord, such as the formation of cystic cavities (Ahuja et al., 2017). Traumatic SCI may cause continued disability and heavy financial burden; however, very few new treatment strategies have been developed to improve the neurologic and physical outcomes of patients after SCI (Karsy and Hawryluk, 2019). Autophagy is an evolutionarily conserved stress-elicited process that sequesters redundant or potentially toxic cytosolic entities within double-membraned vesicles (autophagosomes) and then delivers them to lysosomes for degradation (Galluzzi and Green, 2019). The ubiquitin-like LC3 protein family is involved in autophagosome formation, in which cytosolic LC3-I is conjugated to phosphatidylethanolamine to form LC3-II on the surface of nascent autophagosomes (Runwal et al., 2019). p62 is a predominant mammalian autophagy receptor that is recruited to selected cargoes for autophagic degradation or is degraded as an autophagy substrate itself (Lamark et al., 2017). Many studies have shown a correlation between autophagy and SCI, most of which indicate that autophagy is an important neural recovery mechanism (Liao et al., 2021; Y. Xu et al., 2021).

Ubiquitin protein ligase E3 component N-recognin 1 (UBR1) is a single-subunit E3 ligase of over 200 kDa that catalyzes the ubiquitination of N-degrons for proteasome-dependent protein degradation (M. Pan et al., 2021). UBR1 may participate in motor neuron synchronization and its dysregulation may be related to neuronal development and signaling (Chitturi et al., 2018). Interestingly, our GSE2599 dataset prediction and preliminary experiments revealed a low expression of UBR1 in SCI rats. Meanwhile, ubiquitination and autophagy, two predominant intracellular degradation pathways, are distinct in their mechanisms but inextricably connected in controlling protein quality and maintaining cellular homeostasis (P. Liu et al., 2022). A recently published article demonstrated that UBR1 facilitates the clearance of misfolded MLC1 during Ca^2+^ stress by eliciting p62-mediated selective autophagy (B.B. Wang et al., 2022), which prompted the authors to investigate whether UBR1 is linked to autophagy dysregulation in SCI.

N(6)-Methyladenosine (m6A) is a widespread mRNA internal modification that affects mRNA splicing, stability, or translation, thereby controlling gene expression in various physiological and pathologic processes (Zaccara et al., 2019). Studies have substantiated the alterations in m6A levels after SCI and have shown that many genes with differential m6A modifications are associated with neural regeneration (W. Liu et al., 2021; Xing et al., 2021). METTL3 and METTL14 are two well-characterized human m6A methyltransferases that form complexes that modulate the installation of m6A in mRNA (Oerum et al., 2021). METTL14 has been reported to inhibit EEF1A2 expression in SCI by mediating m6A methylation of EEF1A2 to subsequently block the Akt/mTOR pathway, thereby stimulating apoptosis in spinal cord neurons (Gao et al., 2022). Another study suggested that METTL14-mediated m6A modification in pri-miRNA-375 may induce the apoptosis of spinal cord neurons in SCI by suppressing the expression of RASD1 (H. Wang et al., 2021).

Above evidence stated that METTL14-mediated m6A modification is an important regulatory mechanism underlying SCI-associated neuronal apoptosis. UBR1 is a potential regulator of autophagy, an important neural recovery mechanism after SCI. To date, the m6A modification of UBR1 has yet to be discovered. This study aimed to elaborate on the regulation of METTL14 on UBR1 m6A methylation and more importantly, to determine whether the METTL14/UBR1 axis is implicated in neuronal autophagy and apoptosis in SCI.

## Materials and Methods

### Animals

Animal experiments were conducted with 80 healthy male adult Sprague–Dawley rats (220–250 g) and were approved by the Animal Care and Use Committee of the local hospital. All animals were housed under pathogen-free conditions in a 12/12 h light/dark cycle with a temperature of 18–28°C and humidity of 40–70%, with free access to food and water. Within one week, after the animals had adapted to the environment, they were used for SCI modeling.

### Animal grouping and model establishment

Normal rats with a Basso–Beattie–Bresnahan (BBB) score of 21 out of 21 (baseline) were selected and grouped using a random number table into sham, SCI (1 d), SCI (7 d), SCI (14 d), SCI + lentiviral negative control (LV-NC), SCI + LV-UBR1, SCI + shRNA (sh)-NC, and SCI + sh-METTL14 groups (10 rats per group).

After anesthetization with an intraperitoneal injection of ketamine (80 mg/kg) and xylazine (10 mg/kg), none of the rats had obvious abdominal distention, ascites, discomfort, or pain. Laminectomy was performed at T8–T10 (counting from top to bottom; from the 8^th^ thoracic vertebra to the 10^th^ thoracic vertebra). The spine was immobilized with a stereotaxic device, and the exposed spinal cord was severely injured at the center between T8 and T10 by a 5-g weight dropping freely from a height of 8 cm (Perot et al., 1987; Ray et al., 2000). After surgery, the rats were carefully nursed and fed and their urine was squeezed three times a day until reflex bladder emptying was established. Successful modeling was defined as (1) rapid retraction of the whole body, (2) rapid edema and congestion on the surface of the local spinal cord, (3) intact spinal dura mater, (4) flaccid paralysis of the hindlimbs, and (5) survival. Rats that underwent unsuccessful modeling were replaced. The SCI (1 d), SCI (7 d), and SCI (14 d) groups were killed on first, seventh, and 14th day after modeling, respectively, while the other groups were killed on the 14th day. Most operations in the sham group were the same as those in the SCI group, with no spinal cord contusions. The SCI + LV-NC, SCI + LV-UBR1, SCI + sh-NC, and SCI + sh-METTL14 groups were given lentiviral tail vein injections (once every 3 d, virus titers of 10^8^ TU/ml; GenePharma) 3 d before SCI modeling.

### Cell culture and treatment

PC12 cells (ATCC) were first differentiated in a Petri dish containing β-NGF (50 ng/ml) for 3 d and then cultured in RPMI-1640 medium with 10% fetal bovine serum and 1% penicillin/streptomycin at 37°C in 5% CO_2_. PC12 cells were treated with 560 μm TBHP (Macklin) for 1 h for the simulation of the nerve injury after SCI *in vitro* (Zhou et al., 2020).

### Cell transfection

Lentiviral sh-METTL14, sh-UBR1, sh-YTHDF1, sh-YTHDF2, and/or NCS (GenePharma) were transfected into PC12 cells. The cells were treated with TBHP for 48 h after transfection. The lentiviruses were generated. Specifically, the overexpression (LV-UBR1) or silencing (sh-UBR1: UUGUACAUUCUCUUCUUGCUU; sh-METTL14: AAGUUUCUCUUGUUUCAGCGA; sh-YTHDF1: UUAUUCUCUUGUCCUUUUGUU; sh-YTHDF2: UCAAGUAAGGUUCGAAAUCAU) sequences of the target genes were designed and synthesized. Subsequently, the amplified sequences were inserted into lentiviral vectors (LV6 was used as the overexpression vector and LV-1 as the knock-down vector), and positive clones were confirmed by sequencing to obtain recombinant plasmids. Tool vector plasmids carrying target genes and helper plasmids (for lentivirus packaging) were transfected into 293T cells by GenePharma and cultured for 6 h. The medium was then replaced with complete medium. Seventy-two hours later, cell supernatants were collected, purified, and condensed. The acquired liquid was subjected to a viral titer test at 10^8^ TU/ml.

### BBB score

Motor function of the rat hindlimbs was scored by two professionally trained scientists on the first, seventh, and 14th days after the operation. After adapting to a circular platform with a diameter of 3 m for ∼10 min, the rats were allowed to crawl freely in sequence and each rat was observed for 5 min. The average score given by the two scientists was considered the final BBB score.

### Spinal cord collection and preparation

After anesthetization and cervical dislocation, the rats were confirmed dead without spontaneous breathing or blinking reflex for >5 min. The spinal cord was carefully exposed, the surrounding nerve roots were cut off, and the T8–T10 spinal cord was removed. The contusion site was located at the center part between T8 and T10. The spinal cord from the center to T8 was fixed in 4% paraformaldehyde for 24 h, dehydrated in 70%, 80%, 90%, 95%, and 100% ethanol (1 min each time), cleared twice with xylene (5 min each time), paraffin-embedded, and sectioned (4 μm thick). Last, sections were stored in a −80°C refrigerator for later use.

### TdT-mediated dUTP-biotin nick end-labeling (TUNEL)

The spinal cord sections were placed in an oven for 1 h at 80°C, dewaxed with xylene, hydrated with a gradient of ethanol, and subjected to TUNEL reaction (Roche Diagnostics). Images were captured with an IX-73 fluorescence microscope (Olympus).

Cells were fixed in 4% paraformaldehyde for 30 min and 70% cold ethanol for 15 min, treated with PBS containing 0.3% Triton X-100 for 5 min at room temperature, and incubated with TUNEL detection solution for 1 h at 37°C in the dark. The slides were sealed with an anti-fluorescence quenching solution and observed under a fluorescence microscope. Nuclei were stained with DAPI. The number of cells in five random fields per slide was counted and averaged. The TUNEL^+^/DAPI ratios were then calculated. Cell counts were performed by investigators who were blinded to the group and treatment.

### Hematoxylin-eosin (H&E) staining

Dewaxed and hydrated spinal cord tissue sections were stained with hematoxylin (H8070-5G; Solarbio) for 4 min, differentiated with hydrochloric acid alcohol for 10 s, rinsed for 5 min, and soaked in eosin solution (PT001; Bogoo) for 2 min. After dehydration with gradient alcohol (1 min each time) and transparentization with xylene (twice, 1 min each time), sections were dried, mounted with neutral gum, and observed under an optical microscope (DMM-300D; Caikon).

### Nissl staining

De-paraffinized and hydrated spinal cord tissue sections were stained with toluidine blue at 37°C for 30 min and washed with water for 8 min. After routine dehydration, transparentization, and mounting, the sections were photographed under an optical microscope (200×). Neurons were counted using Image-Pro Plus 6.0, and the average number of motor neurons in five random fields of view per section was calculated.

### Immunofluorescence staining

Spinal cord tissue sections were treated with blocking solution (10% BSA and 2% Triton X-100, 1:1) for 2 h and incubated with primary antibodies (rabbit anti-rat LC3, ab128025, Abcam; mouse anti-rat NeuN, MAB377, Merck Millipore) at 4°C overnight. PC12 cells were fixed in 4% paraformaldehyde-0.1 mol/l PBS solution, treated with 10% normal goat serum for 1 h, and incubated with rabbit anti-rat LC3 (ab128025, Abcam) at 4°C overnight. After PBS washing, the sections were incubated with fluorescein-labeled secondary antibodies (goat anti-rabbit IgG Alexa Fluor 488, ab150077, Abcam; goat anti-mouse IgG Alexa Fluor 647, ab150115, Abcam) at room temperature in the dark for 2 h; the cells were incubated with goat anti-rabbit IgG Alexa Fluor 488 (ab150077, Abcam) at room temperature in the dark for 2 h. Nuclei were stained with DAPI. Images were captured using a fluorescence microscope (TCS SP2, Leica) and imported into ImageJ for processing. The channels were split, and the channels of the two target proteins were reserved. False colors were added to the two channels which were processed using the Coloc 2 plug-in.

### Quantitative RT-PCR (qRT-PCR)

Total RNA was extracted with TRIzol reagent (15596026; Invitrogen). After reverse transcription (RR047A), qRT-PCR was conducted on an ABI7500 instrument (ABI) with 9 μl of SYBR Mix, 0.5 μl of positive primer, 0.5 μl of negative primer, 2 μl of cDNA, and 8 μl of RNase-free dH_2_O under the following conditions: 95°C for 10 min, 45 cycles of 95°C for 15 s, and 60°C for 1 min. Three replicate wells were used for each experiment. Primers (see Table 1 for sequences) were synthesized by Sangon Biotech. The relative expression of the product was calculated by the 2^−ΔΔCt^ method, with β-actin used as the internal reference.

**Table 1:** Primer sequences

Name of primer	Sequences
UBR1-F	GATGCAGATCAGTCTCCGGG
UBR1-R	GTGCTGTGAGACTTCCACGA
METTL14-F	TTACGGCGGC AGCTCCTAGC
METTL14-R	TATTCTTCTA CTTTATCTTC
β-Actin-F	ACCCGCGAGTACAACCTTCT
β-Actin-R	GCCGTGTTCAATGGGGTACT

F, forward; R, reverse.

### Western blotting

The protein concentration in the cell or tissue lysates was measured using a BCA kit (Beyotime) to determine the loading volume. Proteins were mixed with loading buffer (Beyotime), denatured in a boiling water bath for 3 min, separated by electrophoresis (80 V for 30 min and 120 V for 12 h), and transferred onto membranes. Protein transfer was performed in an ice bath at 300 mA for 60 min, followed by 1–2 min of membrane washing. Next, the membranes were soaked in blocking solution for 1 h at normal temperature and incubated with primary antibodies against β-actin (ab8227, 1:2500), METTL14 (ab300104, 1:1000), LC3 (ab128025, 1:1000), Beclin-1 (ab62557, 1:1000), p62 (ab240635, 1:1000), Bax (ab182733, 1:2000), Bcl-2 (ab196495, 1:1000; Abcam), cleaved caspase-3 (9661, 1:1000, Cell signaling Technology), or UBR1 (sc-515753, 1:500, Santa Cruz Biotechnology) on a shaker for 1 h at normal temperature. After washing for 3 × 10 min, the membrane was transferred to a secondary antibody solution (IgG, 1:10,000, Abcam) and incubated for 1 h at room temperature. After color development, the blots were visualized with a chemiluminescence imaging system (Bio-Rad).

### Methylated RNA immunoprecipitation (Me-RIP)

Total RNA was extracted from spinal cord tissue or TBHP-treated PC12 cells by the TRIzol method, from which mRNA was isolated and purified by PolyATtract mRNA Isolation Systems (A-Z5300, A&D. Technology Corporation). Anti-m6A (1:500, ab151230, Abcam) or anti-IgG (1:100, Abcam) antibodies were added to the immunoprecipitation buffer (20 mm Tris, pH 7.5, 140 mm NaCl, 1% NP-40, and 2 mm EDTA) and incubated with Protein A/G magnetic beads for 1 h. The purified mRNA and the bead-antibody complex were added into immunoprecipitation buffer supplemented with RNase inhibitors and protease inhibitors and incubated overnight at 4°C. The RNA was eluted and purified with the use of phenol-chloroform for qRT-PCR analysis.

### Photoactivatable ribonucleoside-enhanced crosslinking and immunoprecipitation (PAR-CLIP)

TBHP-treated PC12 cells were incubated with 200 mm 4-thiopyridine (Sigma-Aldrich) for 14 h and cross-linked via exposure to 0.4 J/cm^2^ radiation at 365 nm. Cell lysates were incubated with METTL14 antibodies (5 and 3 mg) at 4°C. The precipitated RNA was labeled with [g-32-P]-ATP and visualized with autoradiography. Proteins in the precipitate were removed by proteinase K digestion and RNA was extracted for qRT-PCR to detect UBR1 expression.

### RNA stability analysis

PC12 cells were treated with actinomycin D (5 μg/ml, Sigma-Aldrich) to halt mRNA transcription. The stability of the mRNA was evaluated by qRT-PCR at 2, 4, 6, and 8 h after transcriptional inhibition.

### Statistical analysis

Graphs were built with GraphPad Prism 8 (GraphPad Software) and all data were presented as mean ± SD. Comparisons between two groups were performed using *t* test. One-way ANOVA was used to compare three or more independent groups, followed by the Tukey’s multiple comparison test. *p *<* *0.05 was considered statistically significant. All cell experiments were conducted in triplicate.

### Availability of data and materials

The datasets used or analyzed during the current study are available from the corresponding author on reasonable request.

## Results

### UBR1 is poorly expressed in rats with SCI

**Figure 1.**
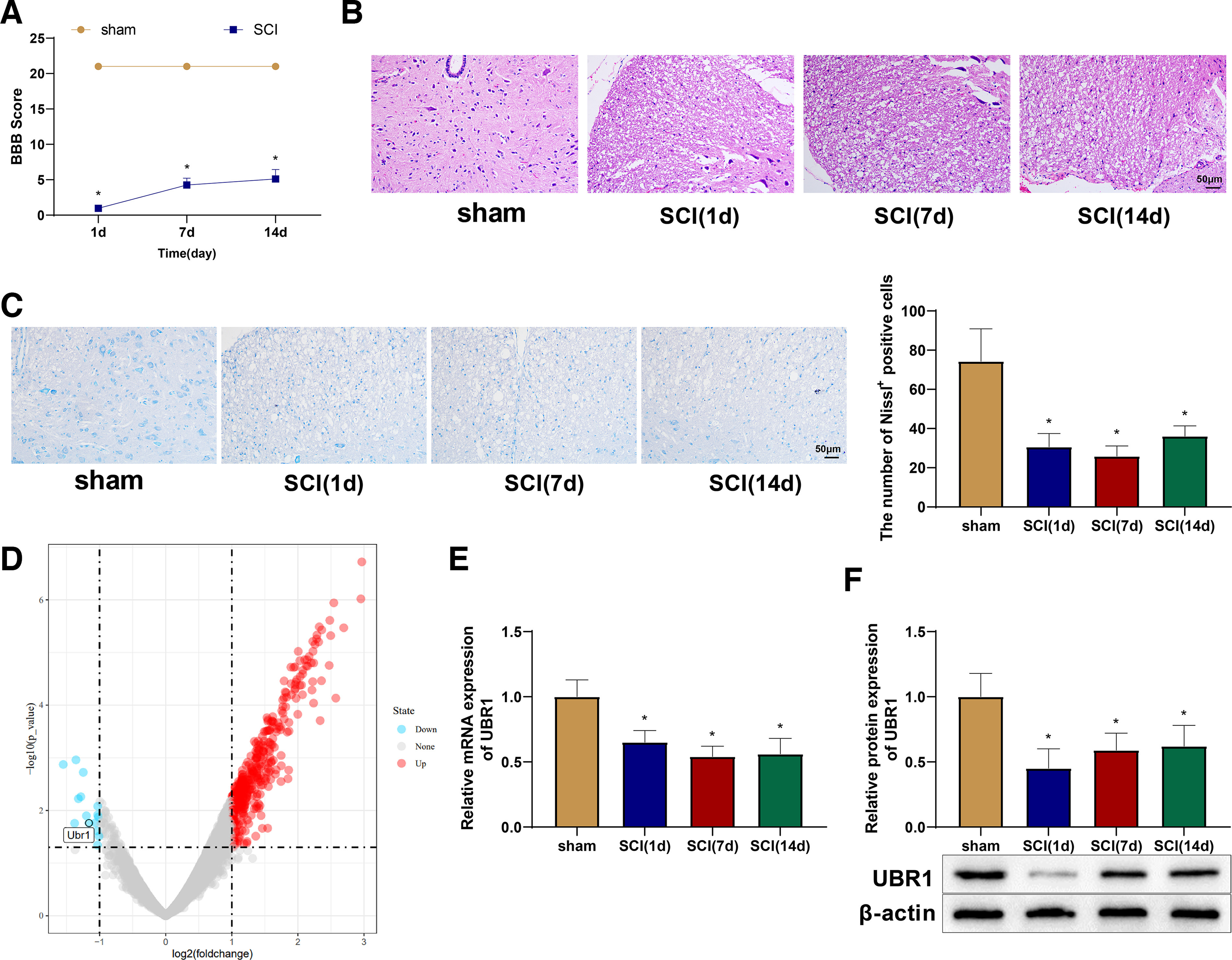
UBR1 expression is poor in SCI rats. ***A***, BBB scores. ***B***, H&E staining of rat spinal cord tissues. ***C***, Nissl staining of rat spinal cord tissues. ***D***, Gene expression in SCI rats in the GEO dataset GSE2599. ***E–F***, qRT-PCR (***E***) and Western blot (***F***) detection of UBR1 expression in rat spinal cord tissues. *means *p *<* *0.05 when the SCI (1 d), SCI (7 d), or SCI (14 d) group was compared with the sham group, respectively. Each group had 10 rats. Panel ***A*** used repeated measures ANOVA, and the rest used one-way ANOVA. BBB, Basso–Beattie–Bresnahan; H&E, hematoxylin-eosin; SCI, spinal cord injury; GEO, gene expression omnibus; qRT-PCR, quantitative real-time polymerase chain reaction; UBR1, ubiquitin protein ligase E3 component N-recognin 1; d, day; ANOVA, one-way analysis of variance.

Rats with a baseline BBB score of 21 were selected for experiments. A rat model of SCI was constructed with a spinal cord contusion which resulted in spastic tail swings and double-hindlimb retraction-like twitches. BBB scores were given to the rats on the first, seventh, and 14th days after SCI modeling, and time-dependent increases were observed, which indicated reductions in muscle strength and tension of the hindlimbs and tails of the rats (Fig. 1*A*). H&E staining revealed that compared with the sham group, the SCI (1 d), SCI (7 d), and SCI (14 d) groups showed disordered spinal cord structures with extensive intracellular and intercellular vacuoles and cavities and a decrease in overall cross-sectional spinal cord areas (Fig. 1*B*). Nissl staining was performed to determine the number of neurons in the spinal cord. Compared with the abundant Nissl bodies and intact tissue structure in the sham group, the number of Nissl bodies was reduced, and the tissue structure was destructed in the SCI (1 d), SCI (7 d), and SCI (14 d) groups (Fig. 1*C*). These findings validated the SCI rat model.

RStudio was used to analyze the GSE2599 dataset from the Gene Expression Omnibus database. This dataset included spinal cord tissue samples from three SCI rats and three normal control rats. High-throughput sequencing analysis was performed on the GSE2599 dataset, and differential analyses of the control and SCI groups were conducted with limma to screen for differentially expressed genes, with the criteria set at |logFC| > 1 and *p *<* *0.05. All poorly expressed genes were ranked according to their fold-changes. Among them, genes with *p *>* *0.05 or previously studied genes were excluded, and the remaining five genes from the top 10 genes were detected by qRT-PCR in animals and cells in the preliminary experiments. UBR1 showed the highest significance and relatively stable expression in both cells and animals. Therefore, UBR1 was selected for subsequent experiments. The preliminary experiments are shown in Extended Data 1. It was predicted through the GSE2599 dataset that UBR1 expression was downregulated in SCI rat tissues (logFC: −1.16, *p *=* *0.02; Fig. 1*D*). Consistently, the expression of UBR1 mRNA and protein, as detected by qRT-PCR and western blotting, respectively, was downregulated in the spinal cord tissues of rats in the SCI group (Fig. 1*E*,*F*).

### Autophagy is activated in spinal cord tissues of rats with SCI

**Figure 2.**
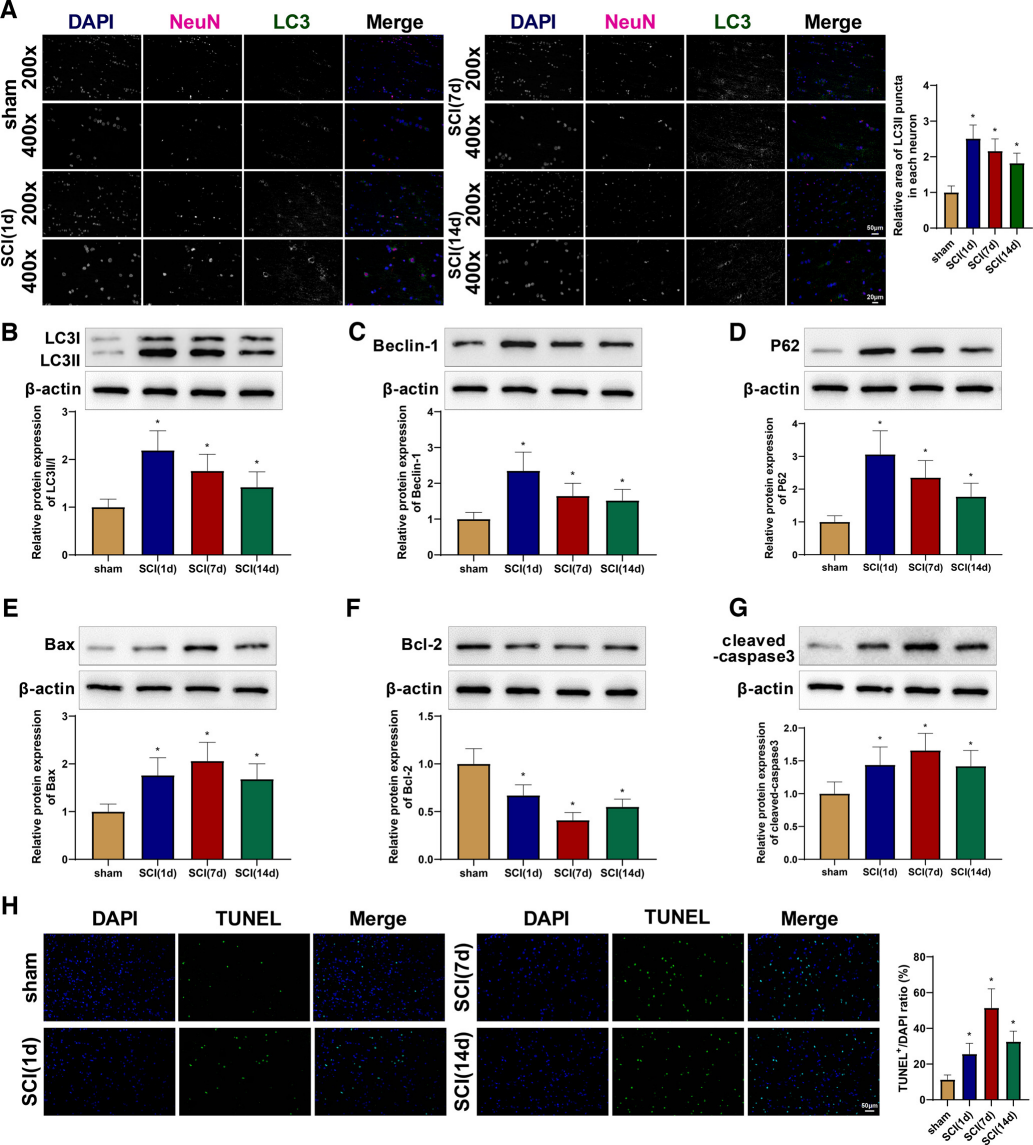
Autophagy is activated in spinal cord tissues of SCI rats. ***A***, Immunofluorescence co-localization of NeuN and LC3 in spinal cord tissues. ***B–D***, Western blot detection of the expression of LC3II/I (***B***), Beclin-1 (***C***), and p62 (***D***) in spinal cord tissues. ***E–G***, Western blot detection of the expression of Bax (***E***), Bcl-2 (***F***), and cleaved caspase-3 (***G***) in spinal cord tissues. ***H***, TUNEL staining to detect the apoptosis of spinal cord tissues. *means *p *<* *0.05 when the SCI (1 d), SCI (7 d), or SCI (14 d) group was compared with the sham group, respectively. Each group had 10 rats. One-way ANOVA was adopted for the group comparisons. SCI, spinal cord injury; Bcl-2, B-cell lymphoma-2; Bcl-2-Associated X (Bax); TUNEL, Terminal deoxynucleotidyl transferase-mediated dUTP nick-end labeling; d, day; ANOVA, one-way analysis of variance.

Colocalization of NeuN and the autophagy-specific protein LC3 by immunofluorescence was used to examine the autophagy of neurons in the spinal cord of SCI rats. The results showed that the SCI (1 d), SCI (7 d), and SCI (14 d) groups had increased expression of LC3II compared with the sham group (Fig. 2*A*). Western blot analysis revealed that the SCI (1 d), SCI (7 d), and SCI (14 d) groups had higher expression of LC3II/I, Beclin-1, and p62 than that of the sham group (Fig. 2*B–D*), suggesting an increase in autophagosomes and autophagy substrates. Notably, the peak autophagy after SCI occurred on postoperative day 1. Moreover, western blotting showed increased protein levels of Bax and cleaved caspase-3 and decreased levels of Bcl-2 in the SCI (1 d), SCI (7 d), and SCI (14 d) groups (Fig. 2*E–G*). Consistently, TUNEL staining of the spinal cord tissues revealed that the apoptosis rate of the SCI rats increased, especially on seventh day after surgery, compared with that of the sham rats (Fig. 2*H*). Collectively, these results demonstrated increased autophagy and apoptosis in the spinal cord of SCI rats.

### UBR1 alleviates SCI, promotes autophagy, and inhibits apoptosis in rats

**Figure 3.**
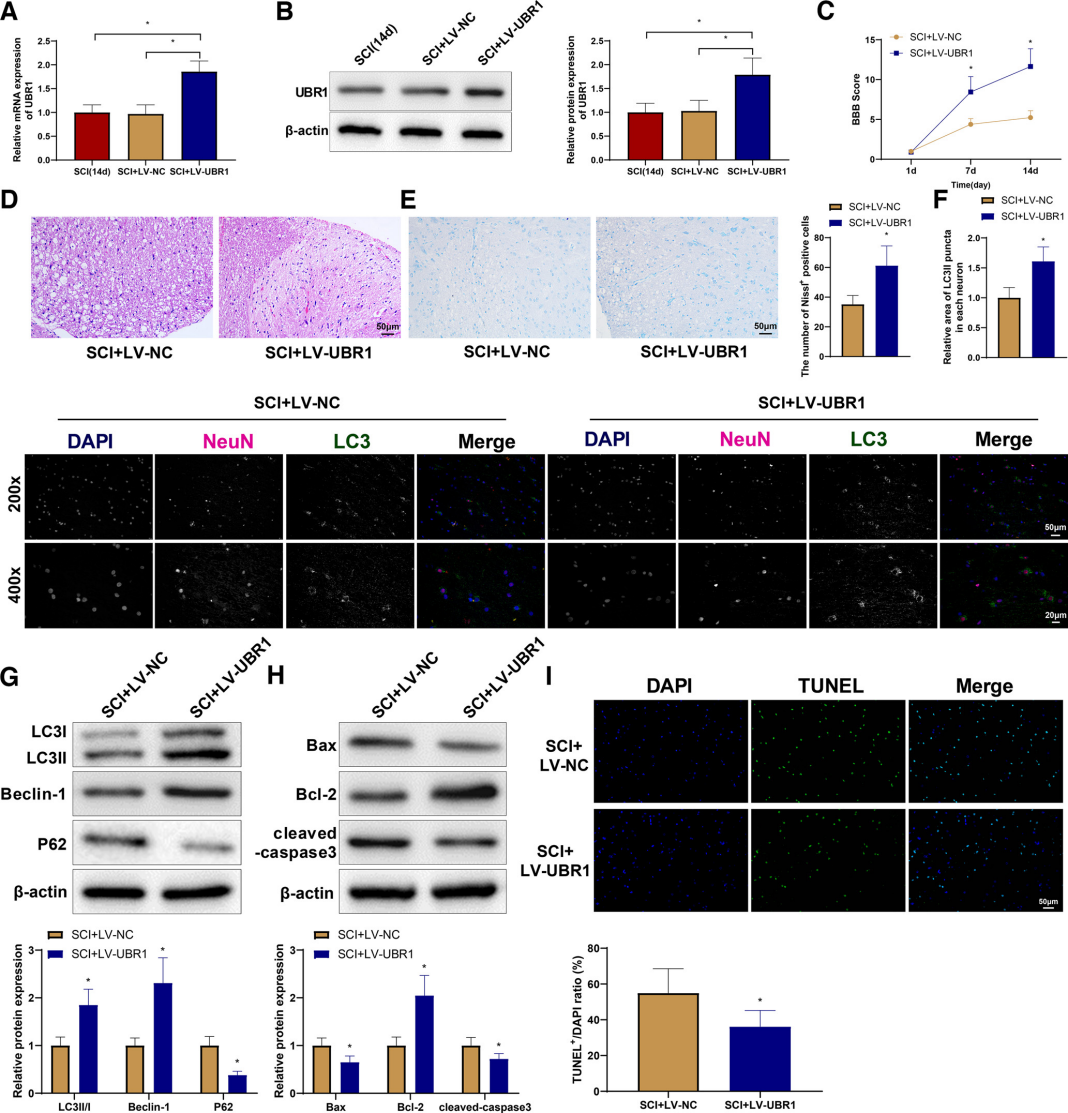
UBR1 alleviates SCI, promotes autophagy, and inhibits apoptosis in rats. qRT-PCR (***A***) and Western blot (***B***) detection of UBR1 expression in rat spinal cord tissues. ***C***, BBB scores. ***D***, H&E staining of rat spinal cord tissues. ***E***, Nissl staining of rat spinal cord tissues. ***F***, Immunofluorescence co-localization of NeuN and LC3 in spinal cord tissues. ***G***, Western blot detection of the expression of LC3II/I, Beclin-1, and p62 in spinal cord tissues. ***H***, Western blot detection of the expression of Bax, Bcl-2, and cleaved caspase-3 in spinal cord tissues. ***I***, TUNEL staining to detect the apoptosis of spinal cord tissues. *means *p *<* *0.05 compared with the SCI (14 d) or SCI + LV-NC group. Each group had 10 rats. Panels ***A*** and ***B*** used one-way ANOVA, panel ***C*** used repeated measures ANOVA, and the rest used *t* test. SCI, spinal cord injury; qRT-PCR, quantitative real-time polymerase chain reaction; UBR1, ubiquitin protein ligase E3 component N-recognin 1; BBB, Basso–Beattie–Bresnahan; H&E, hematoxylin-eosin; Bcl-2, B-cell lymphoma-2; Bcl-2-Associated X (Bax); TUNEL, Terminal deoxynucleotidyl transferase-mediated dUTP nick-end labeling; d, days; LV, lentivirus vector; NC, negative control; ANOVA, one-way analysis of variance.

To study the effect of UBR1 on SCI, we established UBR1-overexpressing SCI and NC rat models via tail vein injections of LV-UBR1 and LV-NC, respectively. The rats were killed 14 d after surgery and spinal cord tissues were collected. Compared with the SCI (14 d) and SCI + LV-NC groups, the SCI + LV-UBR1 group showed higher expression of UBR1 (Fig. 3*A*,*B*). The motor function of the rats was scored on first, seventh, and 14th days after surgery, and the SCI + LV-UBR1 group showed significantly higher BBB scores compared with the SCI + LV-NC group from the seventh day (Fig. 3*C*). Compared with the SCI + LV-NC group, the SCI + LV-UBR1 group still showed extensive cell necrosis in the spinal cord; however, the formation of intracellular and intercellular vacuoles and cavities was limited, and relatively complete cellular structures were observed in the tissues surrounding the cavities (Fig. 3*D*). Moreover, the SCI + LV-UBR1 group exhibited intact cell structures and more Nissl bodies than the SCI + LV-NC group (Fig. 3*E*). Immunofluorescence co-localization demonstrated an increased number of LC3II in the SCI + LV-UBR1 group (Fig. 3*F*). Western blotting showed that the expression of LC3II/I, Beclin-1, and Bcl-2 was increased, while that of p62, Bax, and cleaved caspase-3 was decreased in the SCI + LV-UBR1 group compared with the SCI + LV-NC group (Fig. 3*G*,*H*). The SCI + LV-UBR1 group also showed a lower rate of spinal cord apoptosis than that of the SCI + LV-NC group (Fig. 3*I*). Therefore, the overexpression of UBR1 could improve spinal cord structure, promote autophagy, and inhibit apoptosis in rats with SCI.

### METTL14 upregulates UBR1 m6A methylation to suppress UBR1 expression

**Figure 4.**
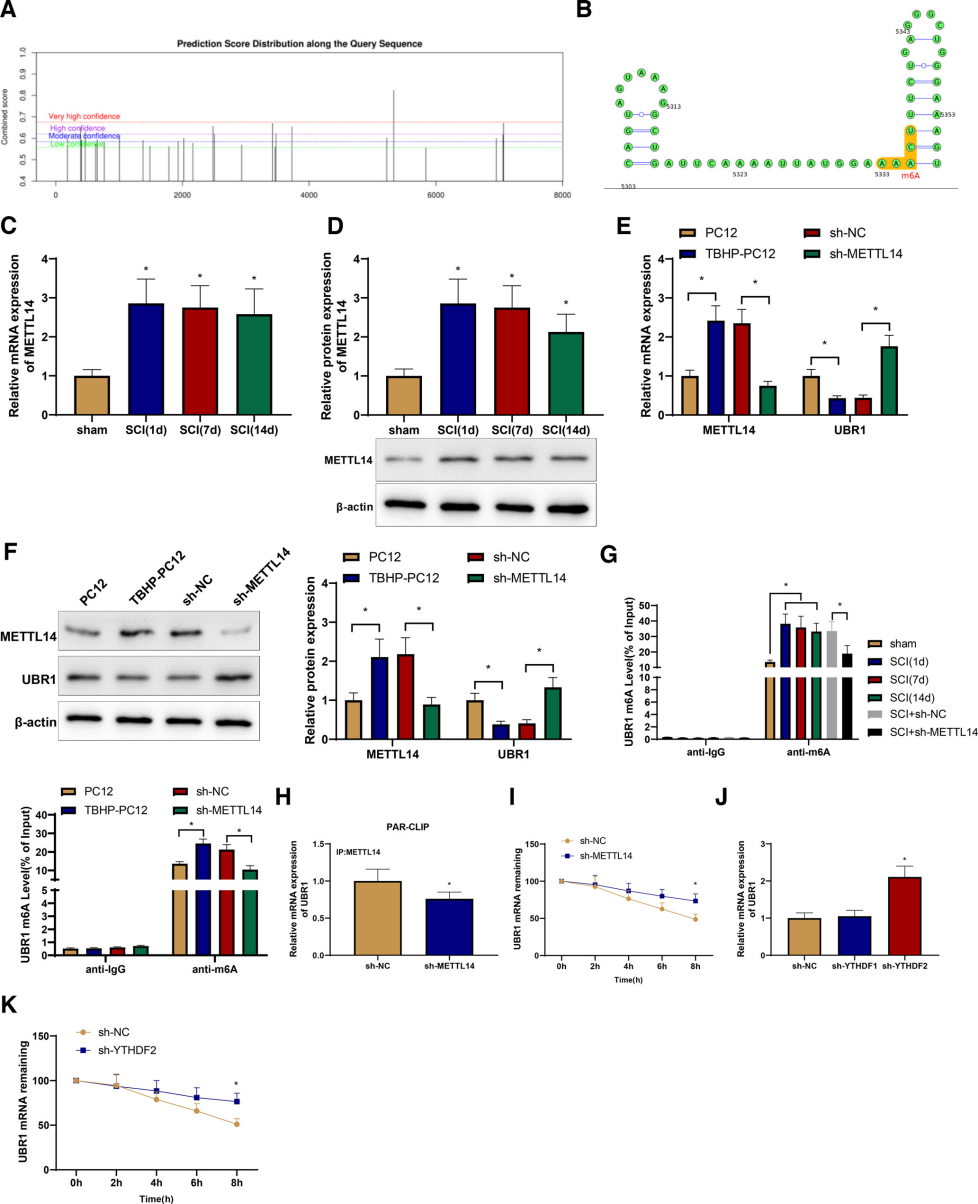
METTL14 upregulates UBR1 m6A methylation to suppress UBR1 expression. ***A***, ***B***, SRAMP analysis of m6A methylation sites in UBR1 mRNA. qRT-PCR (***C***) and Western blot (***D***) detection of the expression of METTL14 in rat spinal cord tissues (*n* = 10). qRT-PCR (***E***) and Western blot (***F***) detection of the expression of METTL14 and UBR1 in TBHP-treated PC12 cells transfected with sh-METTL14 or sh-NC. ***G***, Me-RIP assay to detect the m6A modification levels of UBR1 in SCI rats and TBHP-treated PC12 cells after transfection with sh-METTL14 or sh-NC. ***H***, PAR-CLIP assay to detect the binding of METTL14 mRNA and UBR1 in TBHP-treated PC12 cells transfected with sh-METTL14 or sh-NC. ***I***, Stability analysis of UBR1 mRNA in TBHP-treated PC12 cells transfected with sh-METTL14 or sh-NC. ***J***, qRT-PCR detection of UBR1 mRNA expression in TBHP-treated PC12 cells transfected with sh-YTHDF1, sh-YTHDF2, or sh-NC. ***K***, Stability analysis of UBR1 mRNA in TBHP-treated PC12 cells transfected with sh-YTHDF1, sh-YTHDF2, or sh-NC. *means *p *<* *0.05 compared with the PC12, sh-NC, sham, or SCI + sh-NC group. The animal experiments in panel ***G*** included 10 rats per group. The cell experiments were repeated thrice, and each sample had three replicate wells. Panels ***G*** and ***H*** used *t* test, panels ***I*** and ***K*** used repeated measures ANOVA, and the rest used one-way ANOVA. METTL14, methyltransferase-like protein 14; UBR1, ubiquitin protein ligase E3 component N-recognin 1; mRNA, messenger RNA; m6A, N(6)-Methyladenosine; qRT-PCR, quantitative real-time polymerase chain reaction; n, number; sh, shRNA; NC, negative control; Me-RIP, Methylated RNA immunoprecipitation; SCI, spinal cord injury; TBHP, PAR-CLIP, Photoactivatable ribonucleoside-enhanced crosslinking and immunoprecipitation; ANOVA, one-way analysis of variance.

The online m6A site predictor SRAMP (http://www.cuilab.cn/sramp) showed there were 27 possible m6A methylation sites in UBR1 mRNA and among them, seven were of high confidence and one of extremely high confidence; the latter was in the 3′UTR of UBR1 mRNA (Fig. 4*A*,*B*). Next, we detected the expression of METTL14 in the spinal cord of the model rats with qRT-PCR and western blotting. Compared with the sham rats, SCI rats showed higher expression of METTL14 at different time points after surgery (Fig. 4*C*,*D*). We introduced sh-METTL14 or sh-NC into PC12 cells before TBHP treatment to induce SCI-like cellular damage. TBHP treatment increased METTL14 expression and decreased UBR1 expression in PC12 cells, which was reversed by sh-METTL14 transfection (Fig. 4*E*,*F*). Using the Me-RIP assay, we found that the m6A modification level of UBR1 was markedly augmented in the spinal cord tissues of rats from the SCI group compared with that in the sham group, while silencing METTL14 reduced the m6A modification level of UBR1 in the rat spinal cord. TBHP-treated PC12 cells exhibited an enhancement in the m6A modification level of UBR1 relative to untreated PC12 cells, which was reversed by METTL14 silencing (Fig. 4*G*). Furthermore, the PAR-CLIP assay results showed that silencing METTL14 reduced the amount of UBR1 mRNA pulled down by the METTL14 antibody (Fig. 4*H*), indicating the binding of METTL14 to UBR1. In addition, METTL14 silencing increased the stability of UBR1 mRNA (Fig. 4*I*). YTHDF2, an important reader protein for the m6A modification of RNA, contains a YTH domain that can specifically recognize and bind to m6A-modified RNA and mediate the degradation of the target RNA (J.Y. Wang and Lu, 2021). Based on the concept that METTL14 suppressed the stability of UBR1, we speculated that YTHDF2 is involved in this process. We found that the expression of UBR1 was promoted by sh-YTHDF2 transfection but was not affected by sh-YTHDF1 transfection (Fig. 4*J*), which validated our hypothesis. Furthermore, sh-YTHDF2 transfection enhanced the stability of UBR1 mRNA (Fig. 4*K*). These results suggest that METTL14 represses the expression of UBR1 by promoting the m6A methylation of UBR1 mRNA in TBHP-treated PC12 cells.

### METTL14 promotes SCI, inhibits autophagy, and stimulates apoptosis in rats

**Figure 5.**
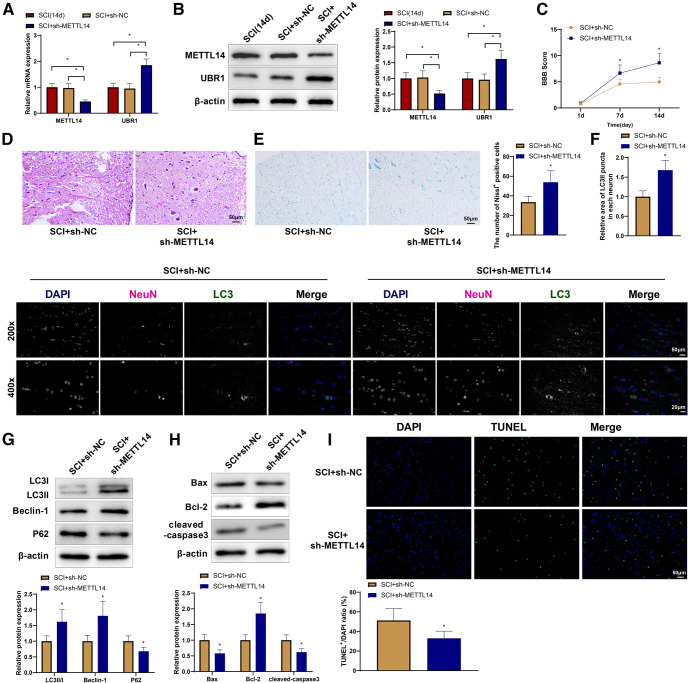
METTL14 promotes SCI, inhibits autophagy, and stimulates apoptosis in rats. qRT-PCR (***A***) and Western blot (***B***) detection of METTL14 and UBR1 expression in rat spinal cord tissues. ***C***, BBB scores. ***D***, H&E staining of rat spinal cord tissues. ***E***, Nissl staining of rat spinal cord tissues. ***F***, Immunofluorescence co-localization of NeuN and LC3 in spinal cord tissues. ***G***, Western blot detection of the expression of LC3II/I, Beclin-1, and p62 in spinal cord tissues. ***H***, Western blot detection of the expression of Bax, Bcl-2, and cleaved caspase-3 in spinal cord tissues. ***I***, TUNEL staining to detect the apoptosis of spinal cord tissues. *means *p *<* *0.05 compared with the SCI (14 d) or SCI + sh-NC group. Each group had 10 rats. Panels ***A*** and ***B*** used one-way ANOVA, panel ***C*** used repeated measures ANOVA, and the rest used *t* test. METTL14, methyltransferase-like protein 14; SCI, spinal cord injury; qRT-PCR, quantitative real-time polymerase chain reaction; BBB, Basso–Beattie–Bresnahan; H&E, hematoxylin-eosin; UBR1, ubiquitin protein ligase E3 component N-recognin 1; Bcl-2, B-cell lymphoma-2; Bcl-2-Associated X (Bax); TUNEL, Terminal deoxynucleotidyl transferase-mediated dUTP nick-end labeling; d, days; sh, shRNA; NC, negative control; ANOVA, one-way analysis of variance.

To study the impact of METTL14 on SCI, we knocked down METTL14 in SCI rats through tail vein injections of sh-METTL14 and killed the rats 14 d after surgery to collect spinal cord tissues. Compared with the SCI (14 d) and SCI + sh-NC groups, the SCI + sh-METTL14 group showed lower METTL14 expression and higher UBR1 expression (Fig. 5*A*,*B*). On day 7 after surgery, the SCI + sh-METTL14 group had significantly higher BBB scores than that of the SCI + sh-NC group (Fig. 5*C*). H&E and Nissl staining revealed that the SCI + sh-METTL14 group had fewer intracellular and intercellular vacuoles and cavities, relatively complete cellular structures, and more Nissl bodies in the spinal cord than that of the SCI + sh-NC group (Fig. 5*D*,*E*). Immunofluorescence colocalization revealed increased expression of LC3II in the SCI + sh-METTL14 group (Fig. 5*F*). Western blotting results demonstrated increased expression of LC3II/I, Beclin-1, and Bcl-2 and decreased expression of p62, Bax, and cleaved caspase-3 in the spinal cord tissues of the SCI + sh-METTL14 group (Fig. 5*G*,*H*). Moreover, spinal cord tissues of the SCI + sh-METTL14 group had fewer apoptotic cells than those of the SCI + sh-NC group (Fig. 5*I*). These results suggest that METTL14 knock-down ameliorates the spinal cord structure, promotes autophagy, and reduces apoptosis in rats with SCI.

### METTL14 promotes apoptosis and inhibits autophagy in TBHP-treated PC12 cells by regulating UBR1

**Figure 6.**
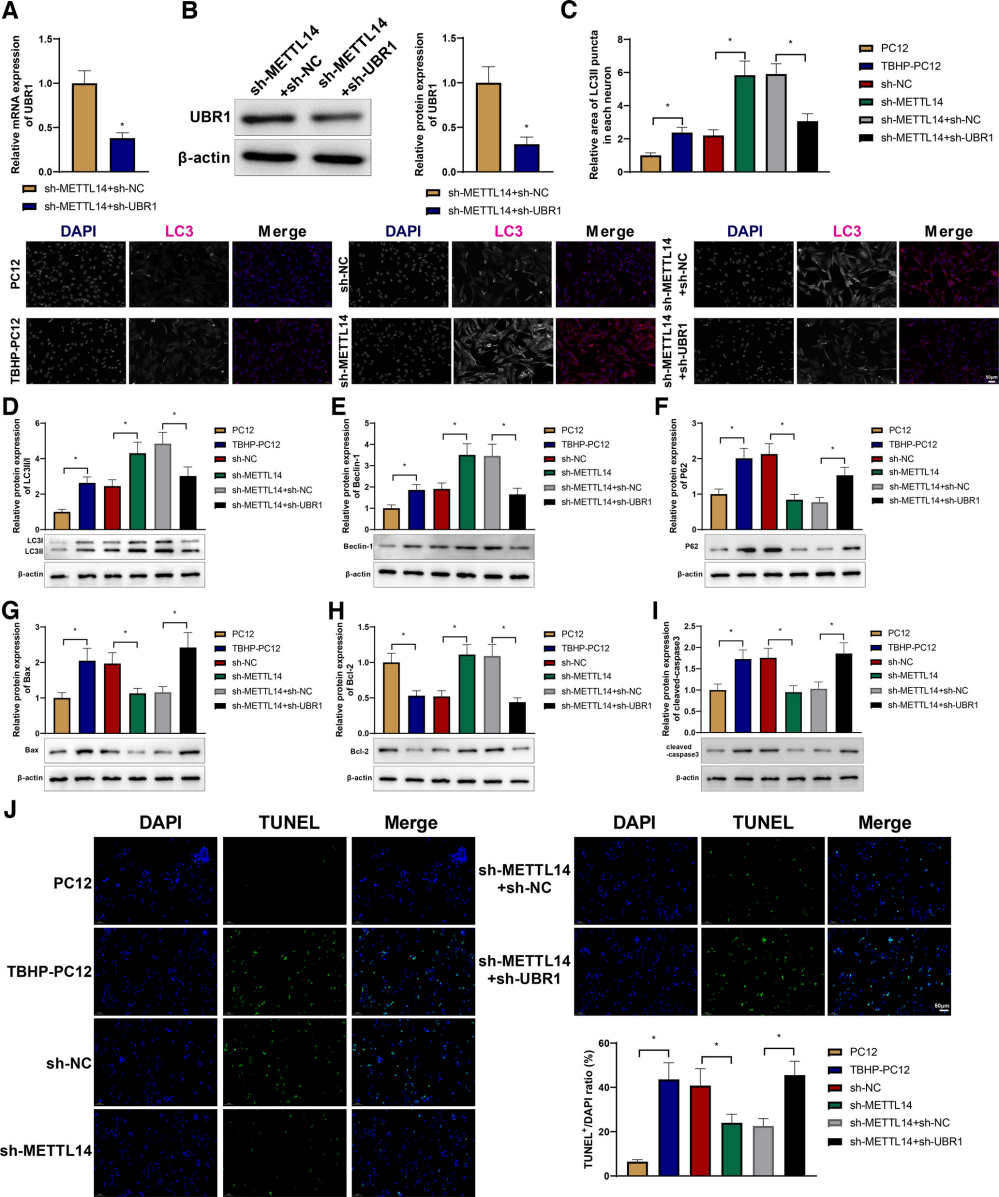
METTL14 promotes apoptosis and inhibits autophagy in TBHP-treated PC12 cells by regulating UBR1. PC12 cells were treated with TBHP 48 h after they were transfected with sh-METTL14 or sh-METTL14 + sh-UBR1. qRT-PCR (***A***) and Western blot (***B***) detection of UBR1 expression in the cells. ***C***, Immunofluorescence detection of LC3 in the cells. ***D–F***, Western blot detection of the expression of LC3II/I (***D***), Beclin-1 (***E***), and p62 (***F***) in the cells. ***G–I***, Western blot detection of the expression of Bax (***G***), Bcl-2 (***H***), and cleaved caspase-3 (***I***) in the cells. ***J***, TUNEL staining to detect the cell apoptosis. *means *p *<* *0.05 compared with the PC12, sh-NC, or sh-METTL14 + sh-NC group. The experiments were repeated thrice, and each sample had three replicate wells. Panels ***A*** and ***B*** used *t* test, and the rest used one-way ANOVA. METTL14, methyltransferase-like protein 14; UBR1, ubiquitin protein ligase E3 component N-recognin 1; qRT-PCR, quantitative real-time polymerase chain reaction; sh, shRNA; Bcl-2, B-cell lymphoma-2; Bcl-2-Associated X (Bax); TUNEL, Terminal deoxynucleotidyl transferase-mediated dUTP nick-end labeling; NC, negative control; ANOVA, one-way analysis of variance.

PC12 cells were treated with TBHP for 48 h after transfection with sh-METTL14 or sh-METTL14 + sh-UBR1. qRT-PCR and western blotting analyses showed that sh-UBR1 repressed the expression of UBR1 in the presence of sh-METTL14 (Fig. 6*A*,*B*). Immunofluorescence of LC3 revealed that TBHP treatment increased LC3II in PC12 cells and that METTL14 knock-down further augmented LC3II expression; however, UBR1 knock-down nullified the increase in LC3II expression caused by METTL14 knock-down (Fig. 6*C*). Western blotting revealed that LC3II/I, Beclin-1, p62, Bax, and cleaved caspase-3 in PC12 cells were upregulated by TBHP treatment, while Bcl-2 was downregulated; after METTL14 knock-down, the expression levels of LC3II/I, Beclin-1, and Bcl-2 increased, while that of p62, Bax, and cleaved caspase-3 decreased; the effects of METTL14 knock-down on autophagy or apoptosis-related proteins were reversed by UBR1 knock-down (Fig. 6*D–I*). As depicted by TUNEL staining, METTL14 knock-down reduced TBHP-induced apoptosis of PC12 cells, which was counteracted by the downregulation of UBR1 (Fig. 6*J*). Together, these results indicate that METTL14 drives TBHP-induced PC12 cell apoptosis and inhibits autophagy by regulating UBR1.

## Discussion

Permanent disabilities are typical consequences of SCI because of the limited ability of repairing in the central nervous system (Courtine and Sofroniew, 2019). In recent years, post-SCI modification of epigenetic landmarks has been shown to be strongly associated with axonal regeneration and neurogenesis, thereby providing a new path for recovery from SCI (Zhang et al., 2020). From an epigenetic perspective, the present study investigated the METTL14-mediated m6A modification of UBR1 mRNA in relation with autophagic changes in SCI. METTL14 may inhibit the expression of UBR1 by catalyzing m6A methylation to inhibit autophagy and promote apoptosis in SCI.

UBR1 is a representative N-recognition protein of the UBR box E3 ligase family which recognizes proteins for degradation via N-degrons and regulates signaling pathways linked to cellular processes such as DNA damage, mitochondrial quality control, apoptosis, and inflammation (Kim et al., 2021). The co-deficiency of UBR1 and UBR2 sensitizes embryonic fibroblasts to UV irradiation-induced apoptosis (Piatkov et al., 2012). The inhibition of Arg/N-degron-dependent ubiquitin ligases, including UBR1, promotes apoptosis in various cancer cell types (Leboeuf et al., 2020). A previous study demonstrated that UBR1 silencing induced by miRNA-H1 encoded from herpes simplex virus 1 results in the accumulation of β-amyloid, a protein associated with neurodegeneration (Zheng et al., 2018). UBR5, another N-recognin, is upregulated by the circular RNA Filip1l in spinal neurons to maintain chronic inflammatory pain (Z. Pan et al., 2019). However, no studies have investigated the expression and function of UBR1 in SCI. In the current study, we detected downregulation of UBR1 in the spinal cord of rats with SCI. More importantly, the overexpression of UBR1 promoted neuronal autophagy and reduced apoptosis in SCI rats, as evidenced by the increased expression levels of LC3II/I, Beclin-1, and Bcl-2 and decreased expression of p62, Bax, and cleaved caspase-3, thereby restoring the motor function of the rats and improving the spinal cord structure. Autophagy is a catabolic process that occurs in response to various stress stimuli and promotes cell survival during energy or nutrient deficiency (Dikic and Elazar, 2018). Hence, this process can be tightly regulated to respond properly to different forms of stress and confer adaptation to the ever-changing environment. Autophagy dysfunction is implicated in the pathogenesis of major human diseases including cancers and neurodegenerative disorders (Klionsky et al., 2021). In SCI, the repair of autophagic flux or activation of autophagy reduces neuronal inflammation, apoptosis, and pyroptosis (Rong et al., 2019; Zhou et al., 2020; Wu et al., 2021; Huang et al., 2022). UBR1 also has the potential to regulate autophagy and mitophagy (Yamano and Youle, 2013; B.B. Wang et al., 2022). These findings, together with ours, suggest that UBR1 alleviates SCI-induced neuronal apoptosis by stimulating autophagy.

Next, UBR1 was found to be regulated by METTL14 which is highly expressed in the spinal cord of rats with SCI. METTL14 catalyzes the m6A modification of UBR1 mRNA and decreases its stability of UBR1 mRNA in collaboration with YTHDF2. METTL14 is indispensable for m6A mRNA methylation and plays an important role in the function of the central nervous system. The METTL14 deletion-induced reduction in m6A impairs dopaminergic neuronal function in the substantia nigra of adult mice (Teng et al., 2021). Knock-out of METTL14 protracts cortical neurogenesis in embryonic mouse brains by reducing m6A tagging of transcripts related to transcription factors, the cell cycle, and neuronal differentiation (Yoon et al., 2017). Peripheral sensory neurons show elevated m6A-tagged transcript levels on injury, which elicit robust axonal regeneration (Weng et al., 2018). m6A methylation is required for normal striatal function, learning, oligodendrocyte maturation, myelination, and embryonic neural stem cell self-renewal (Koranda et al., 2018; Y. Wang et al., 2018; H. Xu et al., 2020). However, elevated METTL14 levels appear to promote neuronal apoptosis after SCI (H. Wang et al., 2021; Gao et al., 2022), which is consistent with our findings that METTL14 knock-down promoted autophagy and reduced apoptosis in the spinal cord of rats with SCI. The effects of METTL14 knock-down were abrogated by UBR1 knock-down in the SCI cell model. Since autophagy can mediate cell survival, the adverse effects of METTL14 may be related to autophagy. Previous studies have shown that METTL14 regulates autophagy in cancer cells (Kong et al., 2020; F. Wang et al., 2021) and inhibited autophagy and stimulates apoptosis and inflammation in injured podocytes by promoting Sirt1 mRNA degradation via m6A modification (Lu et al., 2021). The m6A modification can also reduce testosterone synthesis in Leydig cells by inhibiting AMPK-dependent autophagy (Chen et al., 2021). The epitranscriptomic regulation of autophagy is worth investigating because of its association with human diseases.

In summary, the present study provides compelling evidence that METTL14-mediated m6A modification of UBR1 mRNA modulates neuronal autophagy and apoptosis in patients with SCI. However, the downstream molecules and signaling pathways of UBR1 in SCI remain unclear. Future studies should focus on these unsolved but significant problems.

10.1523/ENEURO.0338-22.2023.ext1 Extended Data 1 Preliminary experiments for the screened differentially expressed genes. (A) qRT-PCR to measure gene expression in spinal cord tissues of rats, n = 3; (B) qRT-PCR to determine gene expression in cells. The experiment was repeated thrice. Download Extended Data 1, DOC file.

## Synthesis

Reviewing Editor: Eran Perlson, Tel Aviv University Decisions are customarily a result of the Reviewing Editor and the peer reviewers coming together and discussing their recommendations until a consensus is reached. When revisions are invited, a fact-based synthesis statement explaining their decision and outlining what is needed to prepare a revision will be listed below. The following reviewer(s) agreed to reveal their identity: Dianna Willis.

In this manuscript, the authors investigate the mechanism by which neuronal autophagy and apoptosis might be regulated following spinal cord injury. They show evidence implicating changes in METTL14-medited methylation of the E3 ubiquitin ligase UBR1. They show that following SCI, the levels of UBR1 are decreased while METTL14 levels are increased and that overexpression of UBR1 or knockdown of METTL14 improved motor function in spinal cord injured rats. This improvement is concurrent with increased autophagy and inhibition of apoptosis. Silencing METTL14 reduced UBR1 methylation, enhancing UBR1 expression. Most interesting, knockdown of UBR1 nullified the increase in autophagy and the decrease in apoptosis induced by the reduction of METTL14. This points to a UBR1 methylation-dependent inhibition of autophagy and promotion of apoptosis, initiated by the activity of METTL14, following SCI. Overall, this manuscript focuses on a topic that is likely to be of interest to journal readers, as the role of autophagy following SCI may be of considerable importance in understanding the pathological mechanism follow damage. This is a second revision of a previously submitted manuscript. The authors have further addressed remaining criticisms through text changes, clarification, and additional data. Overall, these changes have improved that manuscript such that I believe it is suitable for publication after addressing the minor issues below.

1. The staining has been improved throughout. For better visualization given the relatively faint signals, I would suggest showing the staining in monochromatic (i.e., black and white) in the individual channel figures and then in color in the overlay panels. This should improve the ability of the readers to appreciate differences in signals that are then quantified in the graphs.

Furthermore, the analysis is still poorly described and confusing: How Coloc2 was used to obtain the final parameter presented? Coloc2 plugin output is correlation coefficient, not area.

2. “False colors were added to the two channels which were then processed using the Coloc 2 plug-in.” I still don’t understand how Coloc was used. Please elaborate as to how these samples were processed.

3. The DEA analysis is poorly desribed in the results and M&M: It is not helpful to mention R as this is the program language, not the analysis program itself.

More specifically, how was fold-change calculated?

The DEA data file attached has to be available for the reader in the final submission. Authors need to provide clear annotation of the columns, (particularly, what are B & t parameters?)

4. The explanation of choosing UBR1 for further analysis based on the DEA is puzzling. The authors provide no data for the “preliminary experiments” nor any reference for the previously examined, differentially expressed genes in the context of SCI. This part requires major revision to be more coherent, especially since the authors note the interest in UBR1 involvement in SCI in the introduction.

5. The text needs English editing to make it more readable overall.
