# Supplementary material for: Upregulation of UBR1 m6A Methylation by METTL14 Inhibits Autophagy in Spinal Cord Injury
Source: eNeuro. 2023 Jun 2;10(6):ENEURO.0338-22.2023. doi: 10.1523/ENEURO.0338-22.2023 (PMC10241380; doi:10.1523/ENEURO.0338-22.2023)
Supplement: Extended Data 1 — Preliminary experiments for the screened differentially expressed genes. (A) qRT-PCR to measure gene expression in spinal cord tissues of rats, n = 3; (B) qRT-PCR to determine gene expression in cells. The experiment was repeated thrice. Download Extended Data 1, DOC file. [file enu-eN-NWR-0338-22-s01.doc]

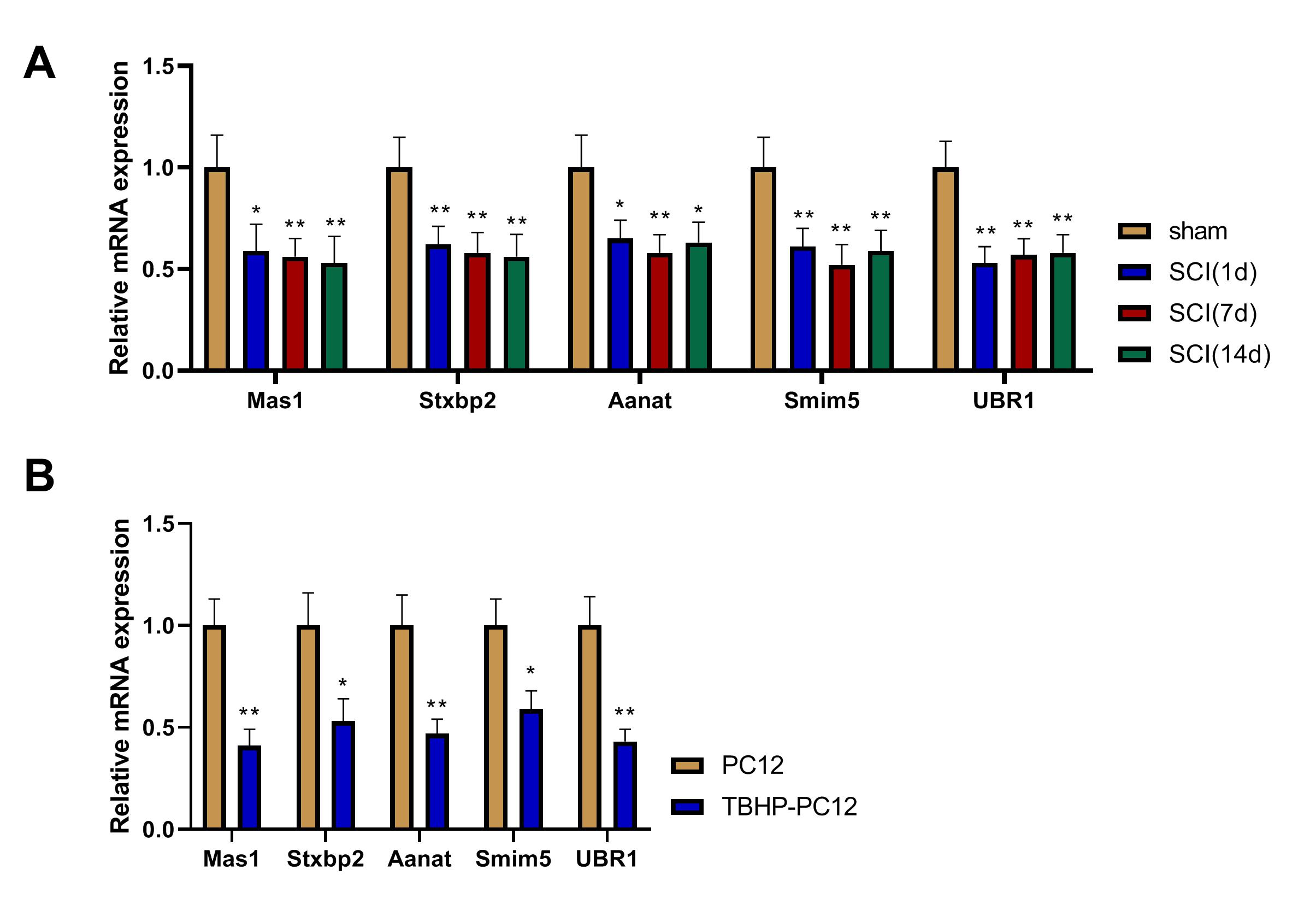


**Extended Data 1** Preliminary experiments for the screened differentially expressed genes

(A) qRT-PCR to measure gene expression in spinal cord tissues of rats, n = 3; (B) qRT-PCR to determine gene expression in cells. The experiment was repeated thrice.
